# Supplementary material for: Are carbon emissions trading and green financial instruments synergistic? -Comprehensive quantitative research based on content analysis
Source: PLoS One. 2024 Mar 7;19(3):e0298601. doi: 10.1371/journal.pone.0298601 (PMC10919601; doi:10.1371/journal.pone.0298601)
Supplement: S1 Data — (ZIP) [file pone.0298601.s001.zip › Data/地级市Z_F工作报告.zip!/╜¡╦╒/╤╬│╟╩╨/└┤╫╘╣1⁄2╓┌║┼í╛┬φ┐╦╩2╛▌═°í┐_2014.docx]

各位代表:

现在,我代表盐城市人民政府向大会作工作报告,请予审议,并请各位政协委员和其他列席人员提出意见。

2013年工作回顾

过去的一年,是我市全面贯彻落实党的十八大、十八届三中全会和习近平总书记系列重要讲话精神,实现经济社会持续健康发展的一年。面对复杂严峻的宏观经济环境,我们在省委、省政府和市委的正确领导下,沉着应对各种风险挑战,积极谋划推动新的发展。经过全市上下共同努力,完成了市七届人大二次会议确定的年度主要目标任务,可望以县为单位在苏北率先实现原省定小康标准,各项工作开创了新局面。

一、强化质量效益导向,经济发展水平实现新提升。针对经济发展中不断出现的新情况、新问题,我们积极创新发展思路,强化运行调节,稳定经济增长,推进转型升级,发展质量和效益明显提高。经济增长稳中有进。预计完成地区生产总值3500亿元,增长12.5%;公共财政预算收入366.8亿元,增长17.3%;规模以上工业增加值1584亿元,增长15.6%;固定资产投资2270亿元,增长22.2%;社会消费品零售总额1160亿元,增长13.4%。8项主要指标增幅位居全省前列,税收占公共财政预算收入比重达82.5%。产业结构明显优化。三次产业结构调整为14.1:47.2:38.7,二三产业比重提高0.5个百分点。推动传统产业改造升级,汽车等四大支柱产业开票销售增长25%。大力发展新能源、节能环保等新兴产业,主营业务收入突破1200亿元,成为国家战略性新兴产业区域集聚发展试点城市。加快30个现代服务业集聚区和100个重点项目建设,服务业投资增长26.6%。新引进银行机构6家,新增贷款余额358亿元。成功举办第六届海盐文化节、第二届汽车博览会和环保产业博览会,荣膺“最美中国•生态旅游目的地城市”。现代农业加快推进。实施农业现代化工程,高效农业总量、增量继续保持全省第一,粮食生产实现“十连增”,再次荣获“全国粮食生产先进市”称号。新发展家庭农场720家、农民专业合作社842家,村级集体经济不断壮大。成功承办第十五届江苏农业国际合作洽谈会。东台市被认定为国家农业产业化示范基地,建湖县建成全国绿色食品原料标准化生产基地,大丰盐土大地农业科技园获批国家级农业科技园区。重点企业转型发展。深入推进“千百十工程”、大企业(集团)培育计划,实施千万元以上技改项目526个,25家企业实现合作重组,11家企业完成上市股改。东风悦达起亚公司产销汽车55万辆,第三工厂建成试生产。悦达集团主营业务收入突破1000亿元,新增德龙、博汇两家销售超50亿元企业。重抓全民创业,出台30条政策措施,在全省率先实施工商注册登记制度改革,加快培育“八大类”创业主体,民营经济蓬勃发展。创新能力不断增强。着力推动科技型中小企业转型升级,建成省级企业技术中心20个,申请专利1.6万件,授权专利5000多件,新申报驰名商标8件,全社会研发投入占地区生产总值比重达1.7%。在上海成功举办第三届沿海发展人才峰会。获批国家可持续发展实验区、国家创新型试点城市、国家信息消费试点市。市开发区软件园获批国家级科技企业孵化器。

二、坚持“三港”联动发展,沿海开放开发取得新突破。始终把沿海开发作为最大最好的机遇,以实施重大项目为抓手,推动“三港”联动、开放发展。港口码头建设取得突破。全面推进“一港四区”建设,盐城港货物吞吐量突破5000万吨。大丰港区石化码头、集装箱码头建成使用,滨海港区10万吨级干散货码头、液体化工码头开工建设,射阳港区万吨级航道正式通航,响水港区5万吨级航道整治工程全线开工。临港产业发展取得突破。列入省沿海开发五年推进计划的78个重点项目累计完成投资2271亿元。江动农业装备机械、中粮肉制品深加工等项目加快推进,中海油LNG、5个海上风电项目获得国家批准,成为国家新能源海水淡化试点城市。载体平台建设取得突破。加快沿海5个经济区发展,建成国家海上风电装备研发中心。成立黄海经济区建设指挥部,编制盐田综合开发利用总体规划、产业规划,经济区主干道路加快建设。国家滩涂综合开发试验区规划编制工作基本完成。盐沪、盐苏、盐常合作共建园区顺利推进。沿海对外开放取得突破。坚持以开放促开发,实施北连央企、南接上海、东向出海战略,成功举办沿海发展央企投资合作洽谈会、日韩沿海招商说明会。新批3000万美元以上项目38个,注册外资实际到账15亿美元。新增进出口企业221家,外贸出口总额38.8亿美元,增长12%。积极打造韩资集聚区、台资新高地,推动开发园区转型升级,19个重点园区业务总收入增长42%,注册外资实际到账占全市比重达70%。盐城综合保税区健康运行。

三、推进城乡统筹建设,生态宜居环境有了新改善。组织修编城市总体规划,加快新型城镇化步伐,推动城乡互动发展。中心城市功能不断提升。实施市区十大类160个城建项目,完成投资242.6亿元。全力推进“八大组团”建设,建军路地下商业街建成营业,金融城一期工程主体基本竣工,新龙广场、金鹰天地、市美术馆等项目快速推进,亭湖、盐都、市开发区新城和城南新区建设取得新的成效。实施“露水增绿”工程,串场河景观带基本建成,推动49家沿河企业转型发展,2200多户居民生活环境和质量得到改善,初步展现出母亲河的美丽景象;大马沟公园、东亭湖公园、天山公园等“十大公园”建成开放,新增城市绿地300多公顷;全面部署市区水环境整治工作,实施河道整治三年行动计划,市区核心区保水活水工程建成使用。完善城市基础设施和功能配套,世纪大道、开放大道南段整治出新,东环路南延段、青年路高架大桥等建成通车,范公路升级改造、盐城内河港开发区码头等加快推进。城市管理水平明显提高。国家园林城市创建通过住建部专家组现场考核和综合评审,正在进行公示。国家卫生城市创建顺利通过省级考核评估。城南新区获批全国首批智慧城市试点城区。县(市)城和重点镇村加快建设。城镇化率达到57%。各县(市)坚持新区建设与老城改造同步推进,城市功能、品质、实力有了新的提升。着力推进沿海5个港城、36个市级城乡统筹试点镇村建设,开展农村环境综合整治,建成961个星级康居乡村、3731个环境整洁村,新增16个省级新农村建设示范村。推动区域供水一体化,解决197万农村居民饮用水安全问题。城乡基础设施逐步完善。临海高等级公路盐城段、332省道大丰东延段、盐都双新大道等建成通车,阜建高速公路、淮盐高速盐城至大丰港段加快推进,连盐铁路盐城段开工建设。南洋机场机坪扩建工程竣工,新开通至厦门、哈尔滨、沈阳、武汉等地航线。泰东河疏浚等工程进展顺利,连申线东台南段航道建成通航,实现了我市航道运输通江达海目标。强化农田水利建设、农业综合开发、土地综合整治,电力、邮政、通信等设施建设取得新进展。生态环境得到有效改善。编制生态文明建设规划,部署生态市建设工作,加强节能减排和环境保护,重点实施80项减排工程,关闭一批重污染企业,新增7个国家级生态镇。启动新一轮清水走廊建设行动,加强大气污染防治,组织绿化重点工程会战,新增造林面积13.2万亩。

四、着力保障改善民生,幸福盐城建设迈出新步伐。坚持民生优先发展,大力实施民生幸福工程。城乡居民收入稳定增长。多渠道扩大就业促进创业,新增城镇就业10.6万人,帮扶7800名就业困难人员再就业,城镇登记失业率2.3%。稳步提高职工工资、城乡低保标准、农村五保供养水平。开展新一轮扶贫开发工作,帮扶16万低收入人口脱贫。城镇居民人均可支配收入24217元,增长10.4%;农民人均纯收入13349元,增长12.2%。社会保障水平稳步提高。开展社会保险全覆盖街道(社区)创建活动,在全省率先同步实施城镇职工和居民大病保险制度,城乡养老、医疗、失业三大保险覆盖率达96%以上。将大市区被征地农民纳入社会保障范围并提高养老金标准,建立残疾人与弱势群体爱心助保制度。扩大住房保障范围,大市区新建各类政策性保障住房39万平方米,发放廉租住房租赁补贴2037户。各项社会事业全面发展。推进各类教育协同发展,新开工建设中小学、幼儿园39所,市一小实验小学校区、聚亨路校区扩建工程加快实施。深化医药卫生体制改革,提高城乡公共卫生服务和新农合保障水平。人感染H7N9禽流感防治工作取得较好成效。实施文化惠民工程,成功举办首届“盐渎风”全民读书月和文化艺术周活动,市图书馆新馆建成开放。13个文化产业集聚区建设初见成效。加快建设城市社区10分钟体育健身圈,青少年校园足球运动获全国一等奖,阜宁县被表彰为全国群众体育先进单位,体育强市创建通过省级验收。加强人口与计划生育工作,人口自然增长率控制在4‰以内。组织开展第三次经济普查工作。注重发挥工会、共青团、妇联、科协等群众团体作用,关心下一代、老龄、残疾人、红十字、慈善等事业进一步发展,供销、气象、防震减灾、住房公积金、新闻出版、广播电视、档案、地方志、人事编制、外事侨务、机关事务管理、保密、援疆等工作取得新的成绩。民生实事工程基本完成。大市区新建6个社区卫生服务中心、9个社区卫生服务站。启动建设市社会福利院二期工程。新增公交车257辆,新增更新出租车695辆。整治老旧小区7个,改造后街巷道65条,新建改造农贸市场8个,更换新建老旧小区信报箱9.6万个。全市新建改造中小学校舍48.4万平方米。新增养老床位1.3万张。为困难群众提供法律援助6500件。兴办民生实事投入多、效果好,受到群众的普遍欢迎。社会大局保持和谐稳定。推进社会管理创新,加强基层基础建设,完善“大调解”工作机制,开展信访积案攻坚化解行动,获评全国新一轮社会管理综合治理优秀市。推进平安盐城、法治盐城建设,坚持公正司法,开展严打专项行动,强化食品药品监管、安全生产、应急管理、消防、民族和宗教事务等工作,连续六年被省评为“平安市”。推进国防动员、国防后备力量和人民防空建设,加强优抚安置、拥军优属、军民共建工作,军政军民关系进一步密切。

五、突出提高行政效能,政府自身建设得到新加强。加快职能转变。实施新一轮行政权力清理下放,57个市级部门下放行政权力事项780项,取消250项。推行政务服务标准化,建立容缺预审制度,全力服务“三重”项目。深化亭湖区盐东镇“扩权强镇”改革试点工作。完善政府工作推进、协调、督查机制,市直部门和单位95项年度重点工作任务基本完成。坚持依法行政。主动向市人大及其常委会报告工作,向市政协通报情况,自觉接受监督。办理人大代表建议180件、政协委员提案235件。认真听取各民主党派、社会团体和人民群众的意见。制定执行重大行政决策程序规定,加强行政执法规范化建设。深化政务公开,加快政府信息资源中心建设,推进行政权力网上公开透明运行。改进工作作风。认真执行中央八项规定和省、市委十项规定,切实加强机关作风建设,市级机关绩效管理、民主评议工作取得新成效。结合贯彻省委关于开展党的群众路线教育实践活动部署,坚持先学先改、即知即改,下半年集中解决了基层群众和企业反映强烈的突出问题160多个。开展省市政风行风热线“四位一体”联动行动,交办问题办结率达93.8%。加强廉政建设。严格落实党风廉政建设责任制,推进惩治和预防腐败体系建设,强化行政监察和审计监督,加大纠风治乱和案件查处力度。坚持艰苦奋斗,制止铺张浪费,树立了政府良好形象。

各位代表,过去一年,全市经济“稳”的基础得到巩固,“进”的态势保持向好,取得的成绩来之不易,这是省委、省政府和市委正确领导的结果,是全市人民迎难而上、开拓进取、团结拼搏的结果。在此,我谨代表市人民政府,向广大干部群众,向人大代表、政协委员和离退休老同志,向市各民主党派、工商联、人民团体和各界人士,向驻盐人民解放军、武警官兵、公安干警、省属驻盐各单位,向所有关心和支持盐城发展的海内外朋友,致以崇高的敬意和衷心的感谢!

各位代表,在肯定成绩的同时我们也清醒地看到,我市经济社会发展中还存在不少矛盾和问题:企业增长面还不宽,部分企业运行困难,经济发展基础不够牢固;产业层次不高,自主创新能力不强,推进转型升级压力较大;沿海开发力量聚合不够,新上重大项目偏少偏重;民生建设欠账较多,基本公共服务保障能力不强,与人民群众的期盼还有较大差距;政府职能转变还不到位,机关作风和效能建设需要进一步加强。对这些问题,我们必须清醒认识、积极应对,在今后工作中切实加以解决。

2014年工作安排

新的一年,我们依然面临着错综复杂的宏观经济环境。盐城经济已进入深化改革攻坚期、结构调整关键期、增长速度换挡期,资源环境要素约束趋紧,各类社会矛盾增多,改革发展稳定的任务十分艰巨。我们一定要科学研判形势,坚定发展信心,进一步增强进取意识、机遇意识、责任意识,抓住用好全面深化改革重大机遇以及江苏沿海发展、长三角一体化发展、“一市一策”等政策机遇,稳中求进,改革创新,推动全市经济社会持续健康发展。

今年政府工作的总体要求是:以邓小平理论、“三个代表”重要思想、科学发展观为指导,深入学习和全面贯彻党的十八大、十八届三中全会和习近平总书记系列重要讲话精神,按照省、市委全会的总体部署,坚持稳中求进工作总基调,以全面深化改革为统领,以提高发展质量和效益为中心,以建设国家可持续发展实验区为抓手,牢牢把握主题主线,深入实施“八项工程”,全力推进经济稳定增长、产业转型升级、沿海开放开发、城乡统筹发展、生态文明建设、民生持续改善、社会和谐稳定,加快建设创业开放生态幸福新盐城,奋力开创全面建设高水平小康社会新局面。

全市经济社会发展主要预期目标是:地区生产总值增长11%左右,公共财政预算收入增长14%以上,固定资产投资增长20%,社会消费品零售总额增长13%,外贸出口总额增长10%,全社会研发投入占地区生产总值比重2.2%左右,城镇居民人均可支配收入增长11%左右,农民人均纯收入增长12%左右,居民消费价格涨幅控制在3.5%左右,城镇登记失业率控制在4%以内,节能减排完成省定目标,人口自然增长率控制在4‰左右。

围绕上述总体要求和目标任务,我们将着力抓好以下十项重点工作:

一、推动全面深化改革,增强经济社会发展动力。认真落实市委关于全面深化改革的实施意见,坚定不移推进重点领域、关键环节改革,释放更多制度红利。

更大力度推进市场化改革。充分发挥市场在资源配置中的决定性作用,毫不动摇鼓励支持民营经济发展,深入实施全民创业三年行动计划,落实全民创业各项政策,拆除一切可以拆除的门槛,营造更加公平的市场环境、更加宽松的政务环境,培育壮大各类市场主体。加快政府投融资平台、国有企业股份制改革,完善现代企业制度,支持社会资本参与资产重组、改制上市,积极发展混合所有制经济。放开投资领域,培育多元化投资主体,引导更多民间资本进入新型城镇化、生产性服务业、社会事业、基础设施等领域,增强发展活力。

抓好可持续发展综合配套改革。把国家可持续发展实验区综合配套改革作为盐城新一轮改革的重大平台,把加强生态文明制度建设作为改革的重点内容,研究制定具体实施方案,为沿海地区可持续发展探索实践、闯出新路。着力构建生态环境建设保护机制,实施最严格的生态空间管制,推动生态资源有序开发、集约利用,抓好沿海生态林建设,实施亚行贷款盐城湿地保护项目,积极创建全国绿化模范城市、国家环保模范城市、生态市。构建推进绿色发展制度体系,坚决把好产业导向关、项目准入关、节能减排关,创建一批资源节约型、环境友好型“两型”园区和企业。构建环境治理长效机制,实施蓝天工程、清水走廊行动,推进城乡环境综合整治,加大污染防治力度,让盐城天更蓝、地更绿、水更清,打造美丽宜居新盐城。

不断深化财税金融体制改革。按照建立现代财政制度的要求,加快实施全面规范、公开透明的预算制度,实行财政预决算和“三公”经费预决算公开。完善市区财税体制,规范政府性基金征收分配秩序。推进“营改增”改革,落实和完善对小微企业的财税支持政策,加大对乡镇、园区培植财源的激励力度。严格政府举债程序,规范债务管理,着力控制、化解地方政府性债务风险。改革财政专项资金使用方式,推行拨改投、拨改贷,组建市创投公司,提高财政资金使用效益,撬动更多的社会资本参与政府主导的公共服务投资领域。深化金融业改革创新,积极引进银行、保险、证券、基金等机构落户金融城,探索组建地方商业银行路径,鼓励发展各类准金融机构,推进村镇银行进乡镇。积极防范金融风险,优化金融生态环境。

二、推动经济稳定增长,努力保持稳中向好态势。积极应对复杂的宏观经济形势,采取更加有力有效措施,促进盐城经济稳中有进、质效提升、行稳致远。

力保企业健康运行。切实加强经济运行分析监测,对重点行业、骨干企业实行一业一策、一企一策服务,加大对中小微企业政策支持力度,及时帮助解决发展难题,促进企业降本增效。健全要素协调机制,推动金融机构扩大信贷投放,帮助企业拓宽融资渠道,加强劳动力供求双方有效对接,协调煤电油气运等要素,保障企业生产需求。加强社会诚信体系建设,清理和规范涉企收费,减少影响企业生产经营的考核达标,营造良好的发展环境。

强力推进项目建设。继续扩大有效投入,增强发展后劲。大力强化专业招商,主攻日韩港台,重点面向世界500强企业、央企、大型民企,着力招引产业链项目、高新技术项目、合作重组项目,推动优势特色产业提档升级、集聚发展,促进现有企业做大做强、裂变发展。组织开展“项目推进年”活动,突出推进“三重”项目,培育一批市场前景好、带动能力强、质量效益高的新增长点。着力优化营商环境,创新服务机制,为加快项目审批、加快建设进度、加快投产达效提供更好服务。

促进消费和出口增长。认真落实促进消费的政策措施,培育消费热点,繁荣商贸流通,开拓农村市场,鼓励电子商务、网络购物、文化休闲、信息、旅游、养老等服务消费,挖掘消费增长潜力。大力发展加工贸易、一般贸易、物流贸易,积极发展市场采购、跨境电子商务、保税贸易等新型贸易方式。着力打造国家级汽车及零部件、石油装备出口基地,培植龙头型出口企业,支持企业在境外设立直销窗口,促进出口贸易快速增长。

三、推动产业转型升级,着力提升区域竞争能力。始终坚持以工兴市第一方略,推进新型工业化与信息化深度融合、先进制造业与现代服务业互动发展。

加快培育新兴产业。围绕建设国家战略性新兴产业区域集聚发展试点城市,实行资源、政策、力量聚焦,推动新兴产业规模发展。新能源产业突出抓好沿海风电场、光伏电站建设,推进海上风电技术研发、整机设备制造和风力发电应用。节能环保产业突出抓好中电投远达环保设备等30个产业链项目,发展先进环保、高效节能、资源循环利用新装备新产品。新能源汽车产业突出抓好电动汽车整车和核心零部件开发,支持新产品推广与应用,积极创建国家新能源汽车示范运行试点城市。电子信息产业突出抓好电子信息网络、电子核心基础技术与器件、新型显示产品、新兴信息服务业。大力发展高端装备、航空装备、海洋产业。

加快提升传统产业。继续实施传统产业改造升级计划,推动集群化、信息化、品牌化发展。汽车产业重点推进东风悦达起亚汽车第三工厂投产达效、汽车研发中心和汽车试验场建设,引进更多的零部件企业,加快汽车零部件、汽车电子产业集聚发展。机械装备产业重点推进江动农业装备制造等项目建设,打造农机装备、石油机械、风电设备、环保机械和泵阀五个百亿元级产业链。纺织服装产业重点推进沙印精纺与织造等项目,发展新型纤维、高档面料、品牌服装等高附加值产品。化工产业重点推进石油化工、石化新材料发展,加快淘汰落后产能,整治安全隐患,关闭能耗较高、污染严重的化工企业。

加快发展现代服务业。以现代服务业集聚区和重点项目建设为抓手,促进服务业发展提速、结构优化、质量提升。实施服务业龙头企业培育计划,重点培植30家规模型、创新型企业。加大现代物流龙头项目招引力度,发展壮大市现代物流园区、市开发区保税物流园区、盐都电商快递园等集聚区,推进物流业标准化信息化现代化建设。大力发展生态旅游和乡村旅游,深度开发丹顶鹤、大纵湖、九龙口、金沙湖等重点景区,麋鹿保护区创建5A级景区。加快发展现代金融、文化、创意、软件、服务外包等新兴服务业态,拉长服务业“短腿”。

加快推进创新发展。突出企业创新主体地位,继续深入实施“千百十工程”,以项目为抓手,加大政策扶持力度,推动企业与行业龙头企业联合、与科研院所联姻、与风投资本联手、与合作伙伴联动发展,实现千家企业技改项目全覆盖,企业重组、上市取得新进展。积极培植一批开票销售超50亿元、超100亿元企业。突出培育科技型中小企业、科技型企业家,推动规模以上工业企业全面建立研发机构。强化人才创新引领作用,实施沿海开发人才集聚、企业家能力提升、高技能人才培育工程,吸引创新资源向盐城集聚,加快建设国家创新型试点城市。

四、推动沿海加快崛起,积极增创新的发展优势。主动对接省沿海开发“六项行动”方案,深入实施市沿海开放发展三年行动计划,完善“三港”联动开发机制,打造活力最强、发展最快的增长板块。

着力提升港口功能。进一步加强“一港四区”资源整合、统筹发展,重点推进6个5万吨级以上码头建设,加快大丰港区10万吨级深水航道、滨海港区中电投煤炭码头、射阳港区物流园区建设,增强港口龙头带动能力。盐城港货物吞吐量达到7000万吨。深化与上海港等国内外知名港口合作,推动盐城港融入上海国际航运中心体系。进一步完善港口集疏运体系,推进淮河入海水道二期工程,加快滨海港区、射阳港区疏港航道建设。加大沿海现代化港城建设力度,提升规划水平,完善基础设施,强化功能配套,优化生态环境,积极发展港口服务业,加快集聚人气,尽快成为县域副中心。

打造临港产业基地。充分发挥沿海战略资源优势,坚持高端引领、生态底线,大力引进临港重大产业项目,建设沿海新型工业基地,努力把沿海建成“经济走廊”、“生态走廊”。利用风光资源,推进中海油LNG、海上风电等项目,加快建设沿海能源产业基地。加强海洋资源综合开发利用,推进新能源海水淡化产业示范、光亚海洋生物科技等项目,加快建设海洋产业基地。依托丰富的农产品资源,推进中粮集团100万头生猪产业化、光明集团100万吨精制糖等项目,加快建设沿海农副产品加工基地。

创新开发体制机制。坚持问题导向,理顺体制、创新机制,激发沿海发展活力。创新投融资机制,积极争取国家和省政策性资金、项目贷款支持,加强与风险投资、股权投资的战略合作,引进社会资本全面进入沿海开发领域。创新合作开发机制,鼓励沿海各地与北京、上海、苏南等地区和大型企业合作,推动市本级深度参与沿海开发,在开放合作中实现借力发展。创新资源管理机制,深化沿海战略资源管控改革,更多利用市场力量推进港口岸线、风光、滩涂、盐田等资源综合利用,促进沿海资源科学有序、高效集约开发利用。

五、推动现代农业发展,提升农业农村工作水平。进一步深化农村改革,实施农业现代化工程,促进农业持续增效、农民持续增收、农村持续发展。

加强现代农业园区建设。推进农业结构调整,发展设施农业、生态农业、外向农业、休闲观光农业。坚持把园区建设作为推进农业现代化的重要抓手,促进园区扩规模、上水平,打造国家沿海现代农业示范基地。加快国家级农业科技园区、城南世界之窗现代农业示范园区、盐龙湖生态示范园等基地建设,开展市级农业园区认定和十佳园区评选活动。加强农业科技创新,推广新品种、新技术、新装备,建立新型农业服务体系,提高粮食生产能力。加大农业投入,推进川东港、中小河流治理等工程,实施黄河故道农业综合开发,加强高标准农田建设。

大力发展农业龙头企业。实施强龙行动计划,以农产品加工集中区为载体,积极引进农业龙头企业,新上一批农业产业化重大项目,扶持现有企业合作重组、培大育强,打造千亿元级农产品加工业。加快市县两级农产品交易市场建设,加强盐沪农产品产销对接,积极发展连锁、配送、网上销售等现代流通业态。健全农业标准体系,大力推进农副产品质量建设、品牌建设,加强食品安全监管,从源头上保障人民群众“舌尖上的安全”。承办好第十六届江苏农业国际合作洽谈会。

构建新型农业经营体系。坚持家庭经营在农业中的基础地位,推进家庭经营、集体经营、合作经营、企业经营等共同发展的农业经营方式创新。加快培育专业种养大户、家庭农场、职业农民等新型农业经营主体,鼓励发展土地流转、股份合作、联耕联种、租赁托管等多种形式的适度规模经营,健全龙头企业和合作组织联农带农机制,提高农业集约经营、规模经营、社会化服务水平,增加农民收入。推进农村集体产权股份合作制改革。深化农村土地制度改革,建立农村产权流转交易市场,开展农村土地确权登记颁证,保障农户宅基地用益物权。以渠北片区、渠南阜宁片区、射阳河畔片区为帮扶重点,建立健全低收入群体增收长效机制。发展壮大农村集体经济,抓好村级组织增收、减负、化债工作。

六、加大城镇化推进力度,促进城乡发展一体化。以人的城镇化为核心,积极稳妥高质量地推进新型城镇化,健全城乡发展一体化体制机制,让城市和乡村渐行渐近。

做大做强中心城市。坚持突出功能配套、改善生态环境、加快项目建设、提升城市品质,更大力度推动大市区建设发展,注重延续历史文脉,突出留住城市记忆,增强中心城市能级。进一步完善城市规划体系,提高规划和建筑设计水平,强化规划执行力。加快“八大组团”建设,实施一批公共配套、社会事业、园林绿化项目,启动开发商业大厦、人民商场、新盐纺、船厂和肉联厂等地块。开工建设火车站换乘中心枢纽捷运系统,提升城市交通集散功能。推进大市区公交一体化,完善公交运行网络,新增公交车300辆,总量突破1000辆。推进大市区供水一体化,实施盐龙湖水厂建设、城东水厂深度处理、盐龙湖水厂与城东水厂水源互通工程,推动供水管网向村居延伸,实现城乡自来水同网同质同服务。推进串场河沿线开发建设,加快建军路改造出新,综合整治8个老旧小区,新建改造7个农贸市场,规划建设盐城静脉产业园、小贩中心。推进公共绿地建设,提升“十大公园”品质,巩固国家园林城市创建成果。加大力度,攻坚克难,继续开展国家卫生城市创建活动。新建50公里城市健身绿色步道。推进城市路网建设,城市快速路一期工程范公路北段建成通车,快速路二期开工建设,完成东北环道路桥梁等工程。坚持重心下移,深化城管体制改革,全面推行城市网格化、扁平化、数字化管理,形成政府监督主导、部门协调运作、市民广泛参与的城市管理新格局。

提升城镇化发展质量。建立城乡统一的规划体系,优化城镇化布局和形态,促进大中小城市、小城镇、农村新型社区协调发展。加快县(市)城发展,着力提升规划设计水平,提升建设管理水平,提升产城融合水平,增强产业承接能力,增强城市创新能力,增强配套服务能力。推进县城城关镇撤镇设街道工作,优化社区布局。突出生态小城镇建设,加大环境整治力度,避免大拆大建;发展镇域特色经济,创造更多就业机会,建设一批工业强镇、商贸重镇、旅游名镇,更加凸显产业特色、乡村特点、生态特质。继续抓好36个市级城乡统筹试点镇村建设,打造331省道、双新大道沿线城乡发展一体化示范带,开展县级城乡统筹试点工作,扩大镇村试点范围,高标准建设新镇区、新园区、新社区。积极探索土地管理、资金保障、住房等领域制度改革,制定放开市区、县(市)城和建制镇落户限制的具体办法,促进农业转移人口市民化。

加快城乡基础设施建设。加大连盐铁路建设力度,推进新长铁路盐城段改造、徐宿淮盐铁路前期工作,力争早日开工建设。加快阜建高速、淮盐高速盐城至大丰港段、233省道、610省道等重点工程建设,实施农村公路提档升级。完成南洋机场飞行区扩建工程。扎实抓好电力、天然气、通信、邮政等基础设施建设。研究制定基本公共服务均等化规划、体系和标准,从群众最需要解决的问题入手,加强基层公共服务机构设施和能力建设,实施新一轮农村实事工程,推进城乡公共交通、区域供水、垃圾和污水处理、消防设施等基本公共服务一体化。

七、加大园区建设力度,更高水平推进对外开放。把园区作为经济建设主战场、对外开放主窗口,开展“载体建设年”活动,推动重点园区转型发展,引领开放型经济体系建设。

推进园区功能完善提升。进一步优化园区发展规划、产业定位,强化产业链招商、专业化招商,培育主导产业、特色产业,鼓励兴建“一区多园”,促进产业集群、企业集聚。着力提升公共服务功能,完善园区基础设施,建立开放共享的信息咨询、储运物流等商务服务平台,加强学校、医院、银行、工业邻里中心等配套设施建设,推进产城互动。着力提升创新驱动功能,加快大学科技园、人力资源服务产业园等创新载体建设,引进高端人才和创新创业团队,鼓励企业科技创业,带动园区创新发展。着力提升对外开放功能,加快打造韩资集聚区、台资新高地,充分发挥盐城综合保税区、大丰港保税仓库作用,建设中坦现代农业示范园,加强与上海自贸区对接合作,深化盐沪、盐苏、盐常合作共建园区,以园区国际化引领开放型经济加快发展。

推进重点园区开发建设。推动市开发区二次创业,加快现代汽车名城、韩资工业园、江动农业装备园、大丰港产业园“一城三园”建设,突破一批重大项目,实现经济增长由汽车主导向多业发展转变。推动盐城高新技术开发区加快升级,发展以新能源装备、高端机床制造、通讯电子为支撑的高新技术产业,争创国家级高新区。推动黄海经济区加快开发建设,研究制定专项支持政策,拉开基础设施框架,招引重大产业项目,打造现代工业新区。推动各县(市、区)开发区加强开放合作,增强发展实力,积极推进有条件的开发园区创建国家级开发区、省级开发区、省级高新区。推动各地中小企业园区集约节约发展,完善功能,筑巢引凤,倾情为想创业的人提供平台,为能创业的人提供服务,为创大业的人提供环境。

八、加大民生保障力度,千方百计增进群众福祉。坚持民生优先、急需先行、主动作为,实施民生优先发展三年行动计划,加快民生幸福八大体系建设,让改革发展成果更多更公平惠及全体人民。

推动社会事业优质均衡发展。抓好教育领域综合改革,提升城乡义务教育水平,新建中小学、幼儿园26所,开工建设城南高级中学,加快教育现代化步伐。深化医药卫生体制改革,全面推进县级公立医院改革,完善基本医疗卫生服务体系,建成市县卫生信息平台,实施市中医院南扩工程,城南医院、市妇幼保健院新院投入使用。全面落实计划生育各项政策,提高出生人口素质。进一步加强精神文明建设,实施文化惠民工程,完善公共文化服务体系,积极开展全民阅读活动,加快书香盐城建设,推动革命遗址修缮保护、市美术馆建设、中波发射台搬迁。优化文化产业园区、特色街区布局,制定支持文化产业发展的政策意见,鼓励有实力的文化企业跨地区、跨行业、跨所有制经营和重组,加快发展“专、精、特、新”中小文化企业,繁荣发展文化产业。广泛开展全民健身运动,加快市建军路全民健身中心、城南体育中心建设。筹建市特殊教育学校教学康复实验综合楼。关心支持老干部、关工委工作,加强人事编制、民族宗教、机关事务管理、外事侨务、科学普及、防震减灾、气象、保密、档案、地方志、红十字、慈善、援疆等工作。

进一步完善社会保障体系。建立经济发展和扩大就业联动机制,加大政府购买公共服务力度,实施就业援助工程,实现更加充分、更高质量就业。健全城乡居民收入增长保障机制,适时适度调整职工最低工资标准,促进企业完善工资决定机制和增长机制,稳步提高各类保险水平,扩大住房公积金制度受益面。完善全市统一的城乡居民社会养老保险制度,实施城乡大病保险制度,并与医疗救助相衔接,建立低收入群体托底保障机制。推广“12349”居家养老服务系统、“6+X”社区综合服务平台,推进市社会福利院二期工程、千鹤湾老年公寓建设,推动乡镇敬老院改造升级。继续实施“爱心助保”制度,完善社会救助体系,更加关爱农村留守儿童、妇女、老人以及残疾人,确保政府救助不遗漏。

集中力量办好民生实事工程。今年继续为民兴办20件实事。全市新建各类政策性保障住房18800套。帮助4000名就业困难人员实现再就业。新建加固中小学校舍30万平方米。实施产业扶贫项目400个,帮扶20万低收入人口脱贫。加大饮用水源地保护力度,解决少数地区饮用水不安全问题。新建改造农村公路700公里、桥梁1300座、无害化卫生户厕10万座。为困难群众提供法律援助6000件以上。加强卫生应急体系建设,大市区新增更换10辆救护车,新建4个卫生服务中心、2个卫生服务站。整治市区核心区9条河道,新建34座城市防洪闸站。

九、加大社会治理力度,确保社会大局和谐稳定。创新社会治理体制机制,深化平安、法治社会建设,确保人民安居乐业、社会安定有序。

改进社会治理方式。坚持系统治理,强化政府主导作用,加强基层基础建设,全面推行“一委一居一站一办”社区管理模式,引导社会各方面有序参与社会管理。坚持依法治理,推进行政依法、公正司法、社会普法、公民守法,建设市县两级法治宣传教育中心、社区矫正管理平台,提高社会治理法治化水平。坚持源头治理,妥善处理和协调各方面利益关系,推动领导干部接访下访常态化,最大限度增加和谐因素。重视乡村组织和城市社区组织建设,加快完善基层治理机构。

加强公共安全管理。完善立体化、现代化社会治安防控体系,完成110指挥系统、社区监控系统升级改造,严防和惩治各类违法犯罪活动。深化安全生产管理体制改革,建立安全隐患排查治理体系和安全预防控制体系,严格落实安全生产责任制。深化食品药品管理体制改革,强化重点环节监管,保障人民群众饮食用药安全。健全防灾减灾应急体制机制,切实提高事前预警、事中救援、事后恢复的综合处理能力。建立健全舆情引导、监管和应对机制,确保信息和网络安全。加强国防动员、国防后备力量、人民防空建设,推动军民融合深度发展。

有效化解社会矛盾。建立畅通有序的诉求表达机制,拓宽社情民意表达渠道。完善矛盾调处机制,加强县镇村调处力量建设,促进人民调解、行政调解、司法调解衔接联动。改革行政复议体制,坚决纠正违法或不当行政行为。深入开展领导干部“三解三促一加强”、“金桥行动”等活动,推行网上受理信访制度,建设“信访零距离”信息化综合服务系统,大力化解信访积案,解决群众合理诉求,把涉法涉诉信访纳入法治轨道。

十、加强政府自身建设,致力打造人民满意政府。加强自身建设是政府工作的永恒主题。我们将坚持问题导向,加快转变政府职能,切实改进政府工作,提高人民群众满意度。

建设创新型政府。坚持把改革创新贯穿政府工作的全过程,贯穿经济社会发展各个领域各个环节。根据新的形势和要求,大力推进思路创新,积极转变发展理念、发展思路、发展方式,不以旧思维解决新问题,不以老办法应对新挑战,推动经济社会发展更有质量、更有效益、更有特色、更可持续。大力推进体制创新,按照省统一部署,扎实稳妥地推进新一轮地方政府机构改革,继续简政放权,深化行政审批制度改革,加大行政审批事项“减、转、放、免”力度,建成新的政务服务大厅,力促“三集中三到位”。大力推进机制创新,更多运用市场化手段集聚资源要素,释放市场活力,同时更好地发挥政府作用。

建设服务型政府。坚持以人为本、执政为民,强化政府公共服务职责,建立健全基本公共服务体系,提供更多更好的公共服务产品。积极推广政府购买服务,凡属事务性管理服务,原则上都要引入竞争机制,通过合同、委托等方式向社会购买。真心服务基层、企业和群众,推动机关干部走入基层“接地气”、深入群众“找灵气”,提高市长热线、市长信箱办理实效,努力做到基层办事无障碍、企业办事无投诉、群众办事无难处。

建设责任型政府。坚持以高度负责的精神做好政府各项工作,强化责任担当,主动作为、奋发进取,多做打基础、利长远的事,遇到困难不退缩、遇到问题不回避、遇到矛盾不绕道,不搞形象工程、政绩工程,努力追求实实在在、没有水分、经济发展质量和效益得到提高又不会带来后遗症的速度。强化责任落实,完善目标管理、督查推进、绩效考核机制,以“钉钉子”精神抓好重点工作、重大项目、重要事项落实,切实增强政府执行力。强化责任追究,严格实施行政问责,重抓机关效能建设,落实首问负责制,及时查处行政不作为、乱作为、慢作为行为。

建设法治型政府。坚持依法行政,严格按照法定权限行使权力、履行职责。认真执行重大行政决策程序,建立健全重大决策社会稳定风险评估机制,努力做到科学决策、民主决策、依法决策。完善行政执法程序,规范自由裁量权。继续深化政务公开,提升行政权力网上公开透明运行质量。自觉接受人大法律监督、工作监督和政协民主监督,深化监察、审计专门监督,重视社会公众监督、新闻舆论监督,认真办理人大代表建议、政协委员提案,提高政府公信力。

建设清廉型政府。坚持廉洁从政、从严治政,建立健全科学有效的权力制约和协调机制,构建决策科学、执行坚决、监督有力的权力运行体系。严格执行《党政机关厉行节约反对浪费条例》和公务接待管理规定,从严控制“三公”经费支出和楼堂馆所建设,控制机构编制和财政供养人员总量。严格执行党风廉政建设责任制,加强公务员队伍教育管理,加大违法违纪案件查办力度,坚决纠正损害群众利益的不正之风。严格执行廉洁自律各项规定,管好自己,带好队伍,树好形象,切实做到干部清正、政府清廉、政治清明。

各位代表,做好新的一年政府工作,面临许多新的形势、新的要求、新的考验,我们深感任务艰巨、责任重大。我们要按照省、市委的统一部署,深入开展党的群众路线教育实践活动,以为民务实清廉为主要内容,突出作风建设,集中解决形式主义、官僚主义、享乐主义和奢靡之风“四风”问题,以反对“四风”的实际成效取信于民,为推动全市经济持续健康发展、全面建设高水平小康社会提供坚强保证。

各位代表,让我们更加紧密地团结在以习近平同志为核心的党中央周围,在省委、省政府和市委的正确领导下,进一步解放思想、锐意改革,抢抓机遇、攻坚克难,为全面完成今年经济社会发展目标任务,谱写好中国梦的盐城篇章而努力奋斗!
